# Development of a Multi-Institutional Prediction Model for Three-Year Survival Status in Patients with Uterine Leiomyosarcoma (AGOG11-022/QCGC1302 Study)

**DOI:** 10.3390/cancers13102378

**Published:** 2021-05-14

**Authors:** Ka-Yu Tse, Richard Wing-Cheuk Wong, Angel Chao, Shir-Hwa Ueng, Lan-Yan Yang, Margaret Cummings, Deborah Smith, Chiung-Ru Lai, Hei-Yu Lau, Ming-Shyen Yen, Annie Nga-Yin Cheung, Charlotte Ka-Lun Leung, Kit-Sheung Chan, Alice Ngot-Htain Chan, Wai-Hon Li, Carmen Ka-Man Choi, Wai-Mei Pong, Hoi-Fong Hui, Judy Ying-Wah Yuk, Hung Yao, Nancy Wah-Fun Yuen, Andreas Obermair, Chyong-Huey Lai, Philip Pun-Ching Ip, Hextan Yuen-Sheung Ngan

**Affiliations:** 1 Department of Obstetrics and Gynaecology, the University of Hong Kong, Pokfulam, Hong Kong; hysngan@hku.hk; 2 Department of Pathology, Pamela Youde Nethersole Eastern Hospital, Chai Wan, Hong Kong; wwc091@ha.org.hk; 3 Department of Obstetrics and Gynaecology, Chang Gung Memorial Hospital, Taoyuan 33305, Taiwan; angel945@cgmh.org.tw (A.C.); sh46erry@ms6.hinet.net (C.-H.L.); 4 Linkou Medical Center, Chang Gung University, Taoyuan 33305, Taiwan; 5 Department of Pathology, Chang Gung Memorial Hospital, Taoyuan 33305, Taiwan; shu922@cgmh.org.tw; 6 Clinical Trial Center, Chang Gung Memorial Hospital, Taoyuan 33305, Taiwan; lyyang@cgmh.org.tw; 7 Pathology Queensland, the Royal Brisbane and Women’s Hospital, Herston, QLD 4029, Australia; m.cummings@uq.edu.au (M.C.); deborah.smith@mater.org.au (D.S.); 8 Centre for Clinical Research, University of Queensland, Herston, QLD 4029, Australia; ao@surgicalperformance.com; 9 Department of Pathology and Laboratory Medicine, Taipei Veterans General Hospital, Taipei 11217, Taiwan; crlai@vghtpe.gov.tw; 10 Department of Obstetrics and Gynaecology, Taipei Veterans General Hospital, Taipei 11217, Taiwan; hylau@vghtpe.gov.tw (H.-Y.L.); msyen@vghtpe.gov.tw (M.-S.Y.); 11 Department of Pathology, the University of Hong Kong, Pokfulam, Hong Kong; anycheun@pathology.hku.hk; 12 Department of Pathology, North District Hospital, Sheung Shui, Hong Kong; lkl549@ha.org.hk; 13 Department of Obstetrics and Gynaecology, Kwong Wah Hospital, Mong Kok, Hong Kong; chanks3@ha.org.hk; 14 Department of Pathology, Kwong Wah Hospital, Mong Kok, Hong Kong; channha@ha.org.hk; 15 Department of Obstetrics and Gynaecology, Queen Elizabeth Hospital, Yau Ma Tei, Hong Kong; liwh1@ha.org.hk; 16 Department of Obstetrics and Gynaecology, Tseung Kwan O Hospital, Tseung Kwan O, Hong Kong; choikm@ha.org.hk; 17 Department of Pathology, Tseung Kwan O Hospital, Tseung Kwan O, Hong Kong; pongwm@ha.org.hk; 18 Department of Obstetrics and Gynaecology, Tuen Mun Hospital, Tuen Mun, Hong Kong; hhf233@ha.org.hk; 19 Department of Obstetrics and Gynaecology, Princess Margaret Hospital, Lai Chi Kok, Hong Kong; h9505645@graduate.hku.hk; 20 Department of Pathology, Princess Margaret Hospital, Lai Chi Kok, Hong Kong; yaoh@ha.org.hk; 21 Department of Pathology, Caritas Medical Centre, Sham Shui Po, Hong Kong; yuenwf2@ha.org.hk; 22 Queensland Centre for Gynaecological Cancer, Royal Brisbane and Women’s Hospital, Herston, QLD 4029, Australia

**Keywords:** uterine leiomyosarcoma, prediction model

## Abstract

**Simple Summary:**

Uterine leiomyosarcoma is an aggressive tumor and the current staging system cannot differentiate the patients into different prognostic groups. This leads to difficulty in predicting the patients’ outcomes and planning for adjuvant therapy. We aimed to develop a prediction model that can predict the chance of survival by the third year. In this article, we had used different statistical tests to identify five readily available clinicopathologic parameters to build the prediction model. Internal validation was performed with satisfactory accuracy. Such a prediction model might help to predict survival outcome, and guide future research on the treatment modality.

**Abstract:**

Background: The existing staging systems of uterine leiomyosarcoma (uLMS) cannot classify the patients into four non-overlapping prognostic groups. This study aimed to develop a prediction model to predict the three-year survival status of uLMS. Methods: In total, 201 patients with uLMS who had been treated between June 1993 and January 2014, were analyzed. Potential prognostic indicators were identified by univariate models followed by multivariate analyses. Prediction models were constructed by binomial regression with 3-year survival status as a binary outcome, and the final model was validated by internal cross-validation. Results: Nine potential parameters, including age, log tumor diameter, log mitotic count, cervical involvement, parametrial involvement, lymph node metastasis, distant metastasis, tumor circumscription and lymphovascular space invasion were identified. 110 patients had complete data to build the prediction models. Age, log tumor diameter, log mitotic count, distant metastasis, and circumscription were significantly correlated with the 3-year survival status. The final model with the lowest Akaike’s Information Criterion (117.56) was chosen and the cross validation estimated prediction accuracy was 0.745. Conclusion: We developed a prediction model for uLMS based on five readily available clinicopathologic parameters. This might provide a personalized prediction of the 3-year survival status and guide the use of adjuvant therapy, a cancer surveillance program, and future studies.

## 1. Introduction

Uterine sarcomas account for 3–8% of all uterine malignancies [1,2], and uterine leiomyosarcoma (uLMS) is the most common histotype accounting for 22.5–44% of all uterine sarcomas [3,4]. The overall 5-year survival rate of uLMS ranges from 18.8 to 68% [5]. But even for stage I–II uLMS, the 5-year survival rate can vary between 40 and 85%, and recurrence rate is 38–50% [3,5,6,7,8,9,10,11,12,13]. The wide range of the survival rates and the high recurrence rate in early-stage disease is likely attributed to the small sample size, pooling of all uterine sarcomas, different diagnostic criteria for uLMS in previous studies, and limitations of the conventional staging system in stratifying patients into different risk groups. In the past, uLMS was staged together with other uterine sarcomas in the same way as for endometrial carcinoma under the 1988 International Federation of Gynecology and Obstetrics (FIGO) staging system [14]. However, uterine sarcomas are a very different disease entity and behave very differently to endometrial carcinomas. Additionally, the biological and clinical characteristics can vary considerably among the different kinds of uterine sarcomas. In 2009, in response to these limitations, the FIGO committee introduced a new staging system specific to each type of uterine sarcoma [15].

However, several studies have shown that both the 1988 and 2009 FIGO systems are not optimal in categorizing patients with uLMS into four clinically meaningful prognostic groups [16,17,18]. Unlike the anatomy-based FIGO systems, the American Joint Committee on Cancer (AJCC) system incorporates tumor grade in addition to the extent of disease in the staging. Zivanovic et al. and Raut et al. compared the 2009 FIGO system with the 6th AJCC system in 2003, and showed that neither system could stratify patients into four clinically meaningful and non-overlapping stages [19,20]. Giuntoili et al. proposed a new staging system comprising tumor size and grade, and showed that its accuracy was not only similar to the 2010 AJCC system [21,22], but was also more accurate in providing prognostic information compared with the two FIGO staging systems [16]. However, the system of Giuntoili has never been externally validated. In an attempt to predict the survival of patients with both low-grade and high-grade uLMS the Memorial Sloan-Kettering Cancer Center derived a nomogram and this was externally validated [23,24]. In these two studies, the proportion of tumors diagnosed as low-grade uLMS were 9% and 12%, respectively. As the histological criteria for low-grade uLMS is controversial and has not been established in the latest WHO classification [4], its diagnosis remains challenging and may not be reproducible. Furthermore, low-grade uLMS consists of a heterogeneous group of tumors with a favorable prognosis, in contrast to high-grade uLMS which has a more aggressive disease course. Hence it is justifiable to separate high-grade from low-grade LMS in risk assessments. Our aim was to develop a simple and meaningful model for predicting outcome for patients diagnosed with high-grade uLMS with cases of low-grade uLMS excluded.

## 2. Materials and Methods

This was a retrospective study comprising more than ten centers from the Asian Gynecologic Oncology Group (AGOG), the Queensland Centre for Gynaecological Cancer (QCGC), and their collaborators in Asia–Pacific region. All centers had obtained ethical approval from their own institutional review board. Patients with a diagnosis of high-grade uLMS in June 1993–January 2014 were identified from the database of each center. The histological features were reviewed by pathologists in the participating centers, and were then centrally reviewed by gynecological pathologists (PPCI, RWCW, CKLL, MC, DS, SHU, and CRL). A minimum of three hematoxylin- and eosin-stained slides per tumor, either as glass slides or as digital slides provided by participating centers, were reviewed. Using the Stanford criteria, the diagnosis of uLMS was made when a uterine smooth muscle tumor showed at least two of the following three features: diffuse moderate to marked nuclear atypia, a mitotic rate greater than 10 MFs/10 HPFs (2.4 mm sq), and tumor cell necrosis [25]. uLMS included in this study were all ‘high-grade’ and met the Stanford and WHO criteria. Tumor diameter was the longest diameter of the tumor that was measured by histological examination, or radiological evaluation if operation was not performed.

Patients’ demographic factors, ancillary investigation findings, treatment details, survival outcomes, and recurrence were retrieved from their clinical records. All the patients were restaged for this study using the 1988 and 2009 FIGO systems as well as the 2010 AJCC staging systems based on the available clinical information. All data collected were summarized with counts (percentages) for categorical variables, mean (+/− standard deviation, SD, and range) for normally distributed continuous variables, or median (interquartile, IQR, or entire range) for other continuous variables. 

### Prediction Model Development

The statistical distribution of each parameter was verified. Those parameters with a skewed distribution were also tested using log transformation. Preliminary screening of the data was performed by univariate Cox regression in prediction of overall survival (OS), which was defined as the time interval from the date of diagnosis to the date of last follow-up or death from any cause, as well as by the univariate binomial regression model using 3-year survival status as the binary outcome (i.e., survival or death). Factors that were used in the univariate analyses consisted of clinical parameters including age, the greatest tumor diameter (and its log transformation), mitotic count (and its log transformation), cervical involvement, parametrial involvement, adnexal spread, retroperitoneal lymph node metastases, and distant metastases (excluding pelvic and retroperitoneal lymph node metastasis), as well as histological features including the presence of satellite nodules, gross and microscopic tumor circumscription, gross features of vascular invasion, microscopic features of lymphovascular space invasion, tumor necrosis, tumor differentiation (spindle cell, epithelioid, myxoid, or mixed), and the presence of nuclear atypia and bizarre cells. The significance of these parameters was further evaluated by multivariate Cox regression and multivariate binomial regression using two different endpoints. Age, tumor diameter, and mitotic count with their log transformation, were continuous variables, while the remainder were categorical variables.

After the above preliminary tests, only those patients with complete data were included to build the prediction models. Multivariate binomial regression models were constructed using the selected predicting variables, and 3-year survival status was used as the primary endpoint which was a binary outcome. Three years was chosen as the cut-off timeframe because the median OS was 3.75 years in one study [23] and it was also about 3.2 years among the 110 patients in our study.

Automated backward model selection method was used to remove all statistically insignificant variables, and the regression model with the lowest Akaike’s Information Criterion (AIC) was chosen. The model was then validated by resampling and internal cross-validation [26,27]. Briefly, the whole dataset was randomly divided into ten equal-sized subsets. Nine of the ten subsets (i.e., learning datasets) were selected and their data were used to construct a separate regression model based on those identified significant variables, and the remaining one subset (i.e., the testing dataset) was used to test the prediction accuracy by comparing the result predicted by the model (i.e., either live or dead at the 3rd year) with the actual survival status. This process was repeated ten times using different combinations of subsets as the learning datasets and testing dataset, i.e., the regression was computed ten times, and ten different prediction values were obtained. The overall accuracy of the final regression model was determined by averaging the ten prediction accuracy values.

Collinearity was assessed by variance inflation factor (VIF) test in the final model. All statistical analyses were conducted using SPSS software version 25 (Statistical Package for the Social Sciences, Chicago, IL, USA) and R version 3.4.3 (version date 30 November 2017, R Foundation, Vienna, Austria).

## 3. Results

### 3.1. Demographic Features

In total, 220 patients with a diagnosis of uLMS were identified. Among them, 19 patients were excluded, where three had no clinical records, one had concurrent lung cancer, two had LMS from extra-uterine origins (one ovarian and one peritoneal), one had controversial features where cervical alveolar rhadomyosarcoma could be a differential diagnosis, and three were re-diagnosed as leiomyoma histological variants (cellular leiomyoma and mitotically active leiomyoma). Nine cases that were considered ‘low-grade’ uLMS or STUMP were also excluded. Among the 201 uLMS patients, the median age at diagnosis was 48.0 years (range 13–83). Almost 70% of the patients belonged to stage I under the 2009 FIGO staging system. The median follow-up duration of our cohort was 36 months (inter-quartile range, 13–77 months). The demographic factors are tabulated in Table 1. 

### 3.2. Univariate and Multivariate Analyses

The results of univariate Cox regression analysis are summarized in Table 2a. Only 179 patients had enough follow-up data for univariate binomial regression analysis and the results are summarized in Table 2b. These results were similar to the results of univariate Cox regression except for lymph node metastasis. All those parameters that were not significant in these univariate analyses were excluded from multivariate analyses, except cervical involvement which was shown to be a clinically significant prognostic indicator [23]. Adnexal metastasis, gross tumor circumscription, and tumor necrosis were excluded because of presence of correlation with other variables.

**Figure 1:**
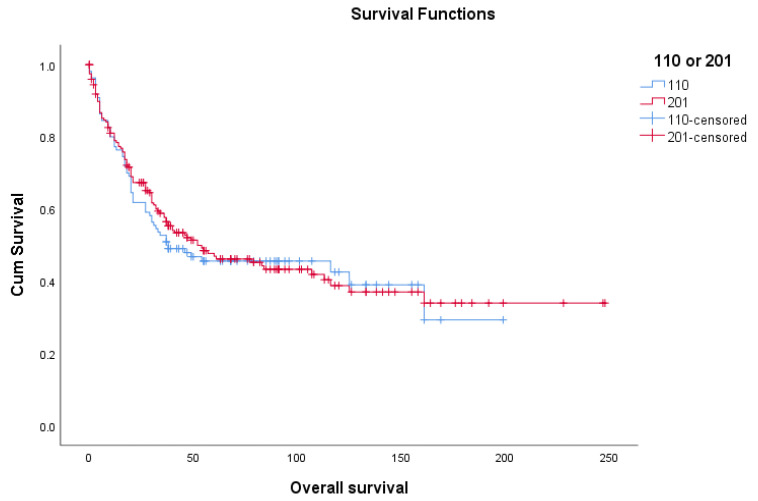
Overall survival of 201 uterine leimyosarcoma patients and 110 patients with complete data.

Nine parameters, including age, log tumor diameter, log mitotic count, cervical involvement, parametrial involvement, lymph node metastasis, and distant metastasis, as well as the microscopic features of tumor circumscription and lymphovascular space invasion were then incorporated into both multivariable Cox regression and multivariate binomial regression analyses in order to identify those independently significant variables that could predict the survival outcomes. Patients with one or more missing values for any of these parameters were excluded, leaving 110 patients in the final analysis model. Their demographic data and overall survival were similar to the 201 patients (Table 1 and Figure 1). The same five variables, including age, log tumor diameter, log mitotic count, distant metastasis, and tumor circumscription were shown to be significantly correlated with the overall survival and 3-year survival status after multivariate Cox regression (Table 3a) and multivariate binomial regression analyses (Table 3b), respectively. 

### 3.3. Construction of Prediction Model

For the construction of the prediction model using 3-year survival status as endpoint, all the nine variables were incorporated into the model. Four parameters that were not statistically significant by the above multivariate analysis were also included because these were clinically meaningful. By using an automated backward selection method, an optimized prediction model consisting of five parameters: age, log tumor diameter, log mitotic count, distant metastasis, and tumor circumscription, with the lowest AIC of 117.56, was constructed. (Table 4). There was no collinearity problem in the final model. Cross-validation-estimated prediction accuracy of this final model was 0.745, indicating the model had an acceptable prediction error. The probability of being alive at three years was able to be predicted by this final binomial regression model using the R software. The database required to construct the model and the steps of execution in the R software are included in the Supplementary Information (Table S1 and Figure S1).

Using this model, for a 45-year-old woman whose log tumor diameter is 1, log mitotic count is 1, the tumor is well-circumscribed with no distant metastasis, her probability of being alive at third years would be 88.4%. Alternatively, for a 60-year-old woman whose log tumor diameter is 1, log mitotic count was 1, but the tumor is infiltrative with distant metastasis, her probability of being alive at third years would be only 16%. 

## 4. Discussion

It is important to have a simple, reliable, and reproducible system that can provide prognostic information to the patients, guide their adjuvant treatment, and identify those who are eligible to join clinical trials so that results can be compared across different risk groups. However, the conventional FIGO staging system for uLMS has many pitfalls due to the rarity of the tumor, lack of prospective data, inconsistency of the diagnostic criteria, pooling all sarcoma subtypes in previous analyses, and an uneven distribution among different stages. For example, although tumor size was shown to be an important prognostic factor in the new FIGO stage I uLMS [28], only 10–23% of the tumors were 5 cm or less [11,19,29,30], and the median size was 7–9 cm [10,13,31,32]. Besides, about half of the patients had stage I disease and other stages were relatively rare [17]. The same phenomenon was seen in the current study, where two-thirds were stage I. Our Kaplan–Meier test also showed that tumor size using 8 cm or 10 cm as cuts-off was a significant prognostic indicator, but not 5 cm. In addition, the practice of pre-operative imaging [33], salpingo-oophorectomy, and pelvic/para-aortic lymphadenectomy, and the use of adjuvant therapy is not standardized across the world [5,12], making validation of any staging system even more difficult. The AJCC system includes tumor grade as part of the staging system. This system considers all retroperitoneal and pelvic sarcomas as deep tumors, and so stages T1a and T2a literally do not exist in uLMS. Besides, other important demographic, clinical and pathological parameters that may predict survival outcomes, such as age and mitotic count, are not taken into account [9,30,31,34,35,36,37,38,39,40]. Therefore, a more personalized assessment is needed.

In recent years, nomograms or scoring systems have become more popular than conventional staging systems in predicting patients’ outcomes in various malignancies. This is because the former incorporate continuous variables, such as age, into the predictive model, and different parameters can be considered at the same time, generating a numeric expression such as survival rate to predict the prognosis of the patients based on their individual characteristics. Some use concordance probability (CP) or index (CI) to measure the prediction accuracy and this can range from 0 (total discordance) to 1.0 (perfect concordance). Most of the CP values ranged from 0.58 to 0.84 [41,42,43,44,45,46,47,48]. There are also several nomogram models for soft tissue sarcoma but none was specific to uLMS [49,50,51,52,53,54]. 

Zhou et al. had used RNA sequencing (RNA-seq) expression profiles and clinical data from The Cancer Genome Atlas (TCGA) to derive a signature of six genes comprising FGF23, TLX2, TIFAB, RNF223, HIST1H3A, and AADACL [55]. The area under the curve values for 12-, 36- and 60-month OS were 0.971, 0.849, and 0.854, respectively. However, this study included only 55 uterine sarcoma samples and the diagnoses were not centrally reviewed. Besides, RNA-seq in the TCGA was performed in different centers and the platforms and the quality of the results might not be standardized. RNA-seq is expensive and is not readily available in many countries, and hence it is difficult to utilize this nomogram globally. The Memorial Sloan-Kettering Cancer Center (MSKCC) developed a nomogram for uLMS using age at diagnosis, tumor size, histologic grade, cervical involvement, loco-regional metastasis (i.e., direct extra-uterine spread or the presence of loco-regional metastases, including regional lymph node metastases), distant metastases, and mitotic index to predict 5-year survival [23]. The bootstrap-validated concordance probability (CP) was 0.65 and this system had been externally validated [24]. Nevertheless, those patients who did not have a hysterectomy due to extensive disease were excluded from the cohort, and this might have potentially led to an over-estimation of the overall survival. Our prediction model incorporated all patients with high-grade uLMS irrespective of the extent of disease, and the histological diagnoses had been confirmed by specialist gynecological pathologists. Therefore, our system would be able to offer an estimation of the three-year survival status. Similar to the MSKCC model, our scoring system did not include the use of adjuvant therapy, because our aim was to provide prognostic information to the physicians as a guide to the suitability of adjuvant therapy. In addition, our data showed that there was no difference in the three-year survival status regardless of the use of adjuvant therapy under the 2009 FIGO staging system (detailed results are not shown).

Our model includes high-grade uLMS only. There are three reasons for this. First, several studies have shown that tumor grade was a prognostic indicator [5,6,16,28,31,56], where high-grade uLMS is usually aggressive and low-grade tumor typically has a favorable prognosis. Second, only about 10% of uLMS are low-grade [23,24,57]. It has also been noted that many of them would be re-classified as other types of uterine mesenchymal tumors after review, as in the study by Zivanovic et al. [23]. Third, it is not uncommon to encounter diagnostic difficulties in differentiating between leiomyoma variants, STUMP, and low-grade uLMS [58,59,60]. Given the fact that the criteria of diagnosis of low-grade uLMS is not clear and may not be reproducible, and since there were only nine cases (8.2%) which fell into this category after review, we decided to exclude them when building the prediction model. 

We incorporated five parameters into the final prediction model after examining all the potential parameters using two different survival endpoints. Age, tumor size, and mitotic count were used as continuous variables because there were virtually no agreed cut-offs for these factors. For example, Garg et al. demonstrated that the 5-year survival rate of patients with tumors of ≤5 cm, >5–10 cm, and >10 cm was 76.6%, 52.9%, and 41.9%, respectively, but there was no difference in the 5-year survival between the latter two groups, suggesting the 5 cm was a better cut-off [28]. On the other hand, a Norwegian study showed that tumor size of >10 cm was an independent prognostic factor in 5-year crude survival (relative hazard 2.7, *p* < 0.001) [11]. Therefore, using it as continuous variable would allow a more precise prediction of survival than using it as a categorical variable.

It is noteworthy that our data showed that tumor circumscription was an independent prognostic factor, and this has not been often reported in the literature. Pelmus et al. showed that macroscopic circumscription was the most significant factor to predict OS among 108 stage I and II uLMS patients (*p* = 0.001) [10]. With more understanding about uLMS, especially in the era of genomic profiling, it is expected that more prognostic indicators will be identified, and our model could provide a platform for further modification in future.

This study was limited by its retrospective nature and some clinical data were missing in their old records. Hence, almost half of the cohort had missed at least one of the nine variables and they could not be tested in the regression models. There was also difficulty in obtaining more paraffin blocks for further pathological and molecular analyses. The data for several immunohistochemical markers for MIB-1, p16, p53, bcl-2, and other hormonal receptors were incomplete, and these could not be evaluated as well. The study was also limited by the relatively small sample size which might make some important parameters less relevant in the univariate and multivariate analyses. However, there were still more than 100 patients with complete data. This study also involved ten different centers in the Asia–Pacific region, and this could potentially avoid bias in the clinical practice in a single center. All the pathological diagnoses and stages were reviewed again in an attempt to have a unified and up-to-date assessment over almost 20 years. Finally, as the study covered almost two decades, some practices might have been evolved. Nevertheless, there was no difference in the survival outcomes of the patients diagnosed between 1993–2003 and 2004–2014 (see Supplementary Information (Table S2 and Figure S2)).

## 5. Conclusions

The FIGO and AJCC systems cannot effectively and reliably stratify patients into four different prognostic groups. In contrast, a nomogram or prediction model can incorporate both continuous and categorical parameters at the same time and provide a personalized risk assessment for individual patients, which may provide information on the use of adjuvant therapy and the frequency and modality of surveillance after treatment. Our prediction model used five parameters including age, tumor size, mitotic count, distant metastasis, and tumor circumscription to predict the 3-year survival status. Such information is readily available from routine investigation and does not involve sophisticated and expensive tests. Although the prognostic parameters were evaluated by different methods and the prediction model was internally validated, the results presented here need to be externally validated by future larger studies.

## Figures and Tables

**Table 1:** Demographics data of the whole cohort and the 110 patients used to build the scoring system.

	Total (201)	Total (110)	*p*-Value
**Regions**			0.486
Hong Kong	143 (71.2%)	71 (64.5%)	
Taiwan	37 (18.4%)	25 (22.7%)	
Australia	21 (10.4%)	14 (12.7%)	
**Age**			0.627
≤50	119 (59.2%)	62 (56.4%)	
>50	82 (40.8%)	48 (43.6%)	
**Median age** (range)	48 (24–83)	49 (24–83)	0.667
**Median tumor size** (cm) (range)	10 (2–37.5)	9.6 (2–37.5)	0.952
**2009 FIGO stage**			0.23
Stage I	139 (69.2%)	75 (68.2%)	
Stage II	22 (10.9%)	13 (11.8%)	
Stage III	13 (6.5%)	2 (1.8%)	
Stage IV	27 (13.4%)	20 (18.2%)	
**Cervical involvement**			
Yes	8 (4.0%)	3 (2.7%)	0.752
No	191 (96%)	107 (97.3%)	
Missing	2		
**Pelvic metastasis ^a^**			0.601
Yes	43 (21.6%)	21 (19.1%)	
No	156 (78.4%)	89 (80.9%)	
Missing	2		
**Distant metastasis ^b^**			0.763
Yes	37 (18.6%)	22 (20%)	
No	162 (81.4%)	88 (80%)	
Missing	2		
**Retroperitoneal lymph node metastasis ^c^**			0.76
Yes	9 (4.5%)	4 (3.6%)	
No	189 (95.5%)	106 (96.4%)	
Missing	3	74	
**Median mitotic index (mitotic figure/HPF)** (range)	14.5 (1–100)	15 (1–100)	0.523
**Primary treatment**			0.854
Surgery alone	88 (43.8%)	46 (41.8%)	
Surgery and adjuvant chemotherapy	49 (24.4%)	28 (25.5%)	
Surgery and adjuvant radiotherapy	44 (21.9%)	26 (23.6%)	
Surgery, adjuvant chemotherapy, and radiotherapy	10 (5.0%)	6 (5.5%)	
Surgery and hormones	2 (1.0%)	0 (0%)	
Surgery, radiotherapy, and hormones	1 (0.5%)	1 (0.9%)	
Others	4 (2.0%)	3 (2.7%)	
Palliative care	3 (1.5%)	0	
**Route of surgery**			
Laparoscopic	10 (5.3%)	3 (2.9%)	0.554
Laparoscopic with vaginal assistance	11 (5.8%)	6 (5.8%)	0.999
Vaginal	1 (0.5%)	1 (1%)	1
Laparotomy	167 (88.4%)	93 (90.3%)	0.614
Total	189	103	
**Bilateral salpingo-oophorectomy (BSO)**			
Yes	144 (71.6%)	79 (71.8%)	0.573
No	55 (27.4%)	31 (28.2%)	
History of BSO	2 (1.0%)	0 (0%)	0.54
Total	201	110	
**Lymphadenectomy**			0.494
Yes	53 (26.4%)	33 (30.0%)	
No	148 (73.6%)	77 (70.0%)	
Total	201	110	
**Survival**			
Number of survival at 3rd year	101 (50.2%)	58 (52.7%)	0.54
Median overall survival (months) (95% CI)	52.0 (22.0–82.1)	38 (0.0–99.5)	0.754
Median progression-free survival (months) (95% CI)	34.0 (8.0–60.0)	29 (0.5–57.5)	0.754
**Follow-up**			
Median follow up duration (months, interquartile range)	36 (12.5–77.5)	37 (16–77)	0.734

^a^ Pelvic metastasis includes metastasis to pelvic peritoneum, cervix, vagina, or parametria. ^b^ Distant metastasis includes metastasis to other organs excluding pelvic peritoneum, cervix, vagina, parametria, and retroperitoneal lymph nodes. ^c^ Lymph node metastasis was determined by either lymphadenectomy or imaging with intraoperative assessment. BSO, bilateral salpingo-oophorectomy; CI, confidence interval; HPF, high power field.

**Table 2:** (**a**) Univariate Cox regression analysis of 201 patients using overall survival as endpoint. (**b**) Univariate binomial regression analysis of 179 patients using 3-year survival status as endpoint.

(**a**)					
**Univariate Cox Regression Analysis (*n* = 201)**					
**Variables**	**Number of Patients**	**Number of Involved Patients #**	**HR**	**95% CI**	***p-*Value**
Age	201		1.05	1.03–1.06	<0.001
Tumor diameter (log)	195		4.69	2.14–10.28	<0.001
Mitotic count (log)	154		3.16	1.70–5.87	<0.001
Cervical involvement	199	8	1.48	0.60–3.65	0.39
Parametrial involvement	199	18	1.92	1.07–3.43	0.029
Adnexal spread *	163	15	2.73	1.44–5.17	0.002
Lymph node metastasis	198	9	0.5	0.14–1.70	0.019
Distant metastasis except lymph node metastasis	199	37	5.18	3.37–7.96	<0.001
Satellite sarcoma nodule (gross feature) *	109	18	1.18	0.59–2.37	0.645
Tumor circumscription (gross feature) *	144	54	1.45	1.10–1.91	0.008
Vascular invasion (gross feature) *	107	5	0.58	0.14–2.39	0.449
Tumor circumscription (microscopic feature)	147	37	0.31	0.16–0.63	0.001
Lymphovascular space invasion (microscopic feature)	146	52	1.89	1.19–3.00	0.007
Nuclear atypia (microscopic feature) *	157	135	1.91	0.91–3.97	0.086
Bizarre cells (microscopic feature) *	153	97	1.03	0.65–1.64	0.902
Tumor necrosis (microscopic feature) *	153	128	2.52	1.16–5.48	0.02
(**b**)					
**Univariate Binomial Regression Analysis (*n* = 179)**					
**Variables**	**Number of Patients**	**Number of Involved Patients #**	**OR**	**95% CI**	***p*-Value**
Age	179		1.06	1.03–1.10	<0.001
Tumor diameter (log)	173		6.84	1.95–26.13	0.004
Mitotic count (log)	135		8.39	2.71–30.21	<0.001
Cervical involvement	177	6	0.64	0.09–3.37	0.61
Parametrial involvement	177	16	3.17	1.10–10.44	0.04
Adnexal spread *	144	131	7.37	1.88–48.83	0.011
Lymph node metastasis	176	8	2.17	0.52–10.85	0.30
Distant metastasis except lymph node metastasis	177	35	9.47	3.91–26.67	<0.001
Satellite sarcoma nodule (gross feature) *	92	16	1.38	0.46–4.12	0.564
Tumor circumscription (gross feature) *	127	50	1.93	1.25–3.05	0.004
Vascular invasion (gross feature) *	92	5	0.31	0.02–2.18	0.301
Tumor circumscription (microscopic feature)	129	33	0.21	0.07–0.53	0.002
Lymphovascular invasion (microscopic feature)	128	44	3.32	1.57–7.24	0.002
Nuclear atypia (microscopic feature) *	137	118	1.71	0.63–5.14	0.31
Bizarre cells (microscopic feature) *	134	97	0.96	0.48–1.95	0.912
Tumor necrosis (microscopic feature) *	133	111	3.00	1.1–9.63	0.043

* These variables were excluded from further analyses. CI, confidence interval; HR, hazard ratio; OR, odds ratio. # Involved patients refer to those who had the features, i.e., patients with cervical involvement, parametrial involvement, adnexal spread, lymph node metastasis, distant metastasis, presence of pathological features including satellite sarcoma nodule, ill-defined tumor circumscription (both as gross and microscopic features), vascular invasion, lymphovascular space invasion, nuclear atypia, bizarre cells, or tumor necrosis.

**Table 3:** (**a**) Multivariate Cox regression analysis (*n* = 110). (**b**) Multivariate binomial regression analysis for constructing prediction model (*n* = 110).

(**a**)			
**Multivariable Cox Regression Analysis (*n* = 110)**			
**Variables**	**HR**	**95% CI**	***p*-Value**
Age	1.04	1.01–1.06	0.002
Tumor diameter (log)	6.28	1.70–23.2	0.006
Mitotic count (log)	3.38	1.54–7.45	0.002
Cervical involvement	1.35	0.32–5.79	0.686
Parametrial involvement	0.58	0.20–1.66	0.31
Lymph node metastasis	0.57	0.16–1.97	0.373
Distant metastasis except lymph node metastasis	4.06	1.74–9.50	0.001
Tumor circumscription (microscopic feature)	0.32	0.14–0.72	0.006
Lymphovascular invasion (microscopic feature)	1.08	0.60–1.92	0.804
(**b**)			
**Multivariate Binomial Regression (*n* = 110, with 62 (56.3%) Deceased)**			
**Variables**	**OR**	**95% CI**	***p*-Value**
Age	1.05	1.01–1.11	0.035
Tumor diameter (log)	11.8	1.48–122.79	0.027
Mitotic count (log)	9.78	2.01–58.82	0.007
Cervical involvement	0.12	0–3.75	0.317
Parametrial involvement	0.93	0.14–8.11	0.943
Lymph node metastasis	0.28	0.01–8.43	0.398
Distant metastasis except lymph node metastasis	6.59	1.25–55.01	0.043
Tumor circumscription (microscopic feature)	0.25	0.07–0.83	0.03
Lymphovascular invasion (microscopic feature)	1.67	0.58–4.83	0.339

CI, confidence interval; HR, hazard ratio.

**Table 4:** Final binomial regression prediction model (*n* = 110).

Finalized Binomial Regression Model (*n* = 110)			
Variables	OR	95% CI	*p*-Value
Age	1.05	1.01–1.11	0.026
Tumor diameter (log)	9.97	1.37–91.21	0.03
Mitotic count (log)	10.84	2.31–62.52	0.004
Distant metastasis except lymph node metastasis	5.1	1.36–25.35	0.025
Tumor circumscription (microscopic feature)	0.25	0.07–0.81	0.027

CI, confidence interval; HR, hazard ratio.

## Data Availability

The data presented in this study are available on request from the corresponding authors.

## Supplementary Materials

The following are available online at https://www.mdpi.com/article/10.3390/cancers13102378/s1, Figure S1: Supplementary material for R execution, Figure S2:Median progression-free survival and overall survival of the original 201 cohort and the 110 patients with complete information on the nine parameters, stratified according to the years of diagnosis Table S1: Database for building the prediction model, Table S2: Survival data of the original 201 cohort and the 110 patients with complete information on the nine parameters, stratified according to the years of diagnosis.
