# Supplementary material for: Development of a Multi-Institutional Prediction Model for Three-Year Survival Status in Patients with Uterine Leiomyosarcoma (AGOG11-022/QCGC1302 Study)
Source: Cancers (Basel). 2021 May 14;13(10):2378. doi: 10.3390/cancers13102378 (PMC8155866; doi:10.3390/cancers13102378)
Supplement: Supplementary file 1 [file cancers-13-02378-s001.zip › Figure S1_Supplementary material for R execution.pdf]

R coding supplementary

1. A. Multivariate binomial regression

```
> t3<-read.csv("D:/Dr Ka Yu/sarcoma/manuscript/Database for building prediction model.csv")
> t3.complete<-t3[complete.cases(t3),]


t3.complete.binomial <-
glm(ThreeYearSurvival~Age+log_tum_dia+log_MF+Cervix+Parametrium+LymphNode+Distant
_exceptLN+MF_VascularInvasion+MF_Circumscription, family=binomial, data=t3.complete)
> summary(t3.complete.binomial)
```

B. multivariate cox regression

```
t3.complete.survival<-
Surv(OS,Dead)~Age+log_tum_dia+log_MF+Cervix+Parametrium+LymphNode+Distant_except
LN+MF_VascularInvasion+MF_Circumscription
> t3.cox<-coxph(t3.complete.survival, data=t3.complete)
> summary(t3.cox)
```

2. Backward selection

```
Full<-
glm(ThreeYearSurvival~Age+log_tum_dia+log_MF+Cervix+Parametrium+LymphNode+Distant
_exceptLN+MF_VascularInvasion+MF_Circumscription, family=binomial, data=t3.complete)
> Backward<-step(Full, direction="backward")
```

3. Final model

```
t3f.complete.binomial<-
glm(ThreeYearSurvival~Age+log_tum_dia+log_MF+Distant_exceptLN+MF_Circumscription,
family=binomial, data=t3.complete)
```

4. Model validation

```
install.packages("DAAG")
> require(DAAG)
cv.binary(t3f.complete.binomial,nfolds = 10)
```

5. 3 year survival prediction of the two examples

```
> newdata <- data.frame(Age=45,log_tum_dia=1,log_MF=1,Distant_exceptLN=0,
MF_Circumscription=1)
predict(t3f.complete.binomial, newdata, type="response")

newdata2 <- data.frame(Age=60,log_tum_dia=1,log_MF=1,Distant_exceptLN=1,
MF_Circumscription=0)
> predict(t3f.complete.binomial, newdata2, type="response")
```
