# Supplementary material for: Development of a Multi-Institutional Prediction Model for Three-Year Survival Status in Patients with Uterine Leiomyosarcoma (AGOG11-022/QCGC1302 Study)
Source: Cancers (Basel). 2021 May 14;13(10):2378. doi: 10.3390/cancers13102378 (PMC8155866; doi:10.3390/cancers13102378)
Supplement: Supplementary file 1 [file cancers-13-02378-s001.zip › Figure S2_Survival curves of the patients stratified by the years of diagnosis.pdf]

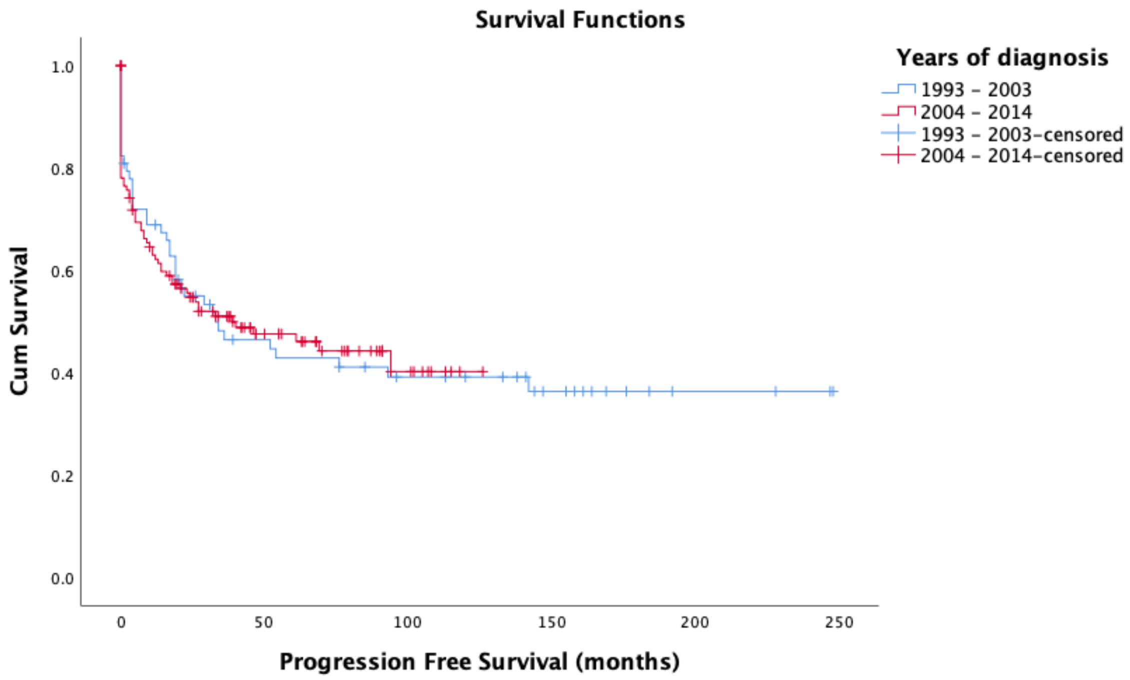

a. Progression-free survival of 201 patients stratified by the years of diagnosis

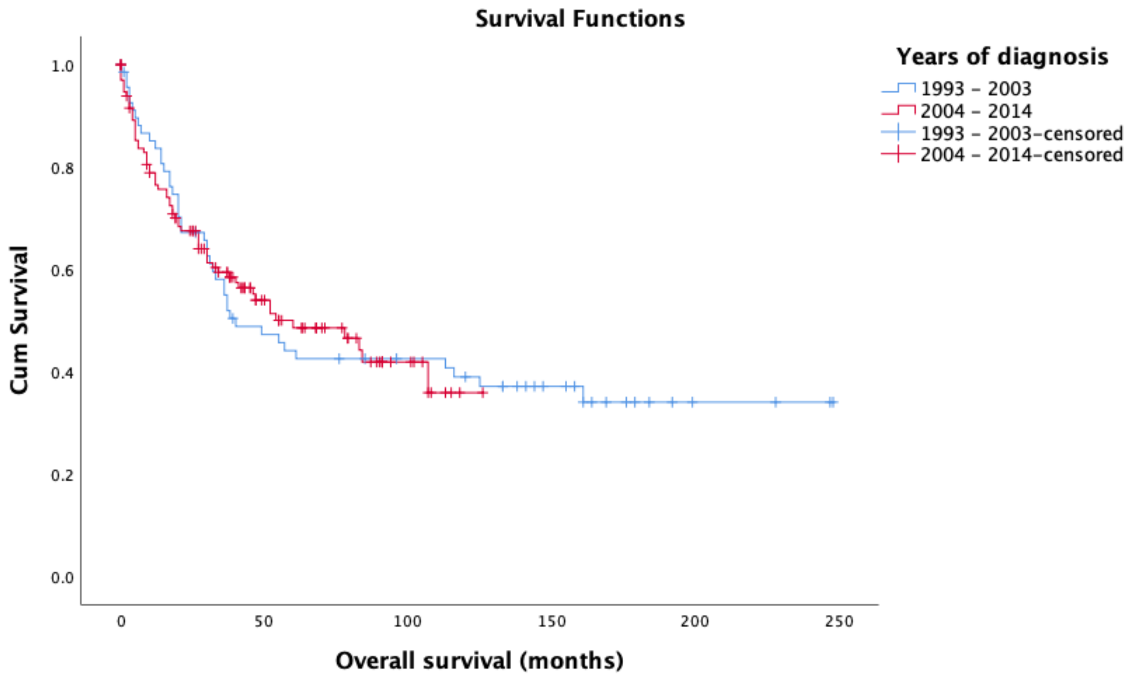

b. Overall survival of 201 patients stratified by the years of diagnosis

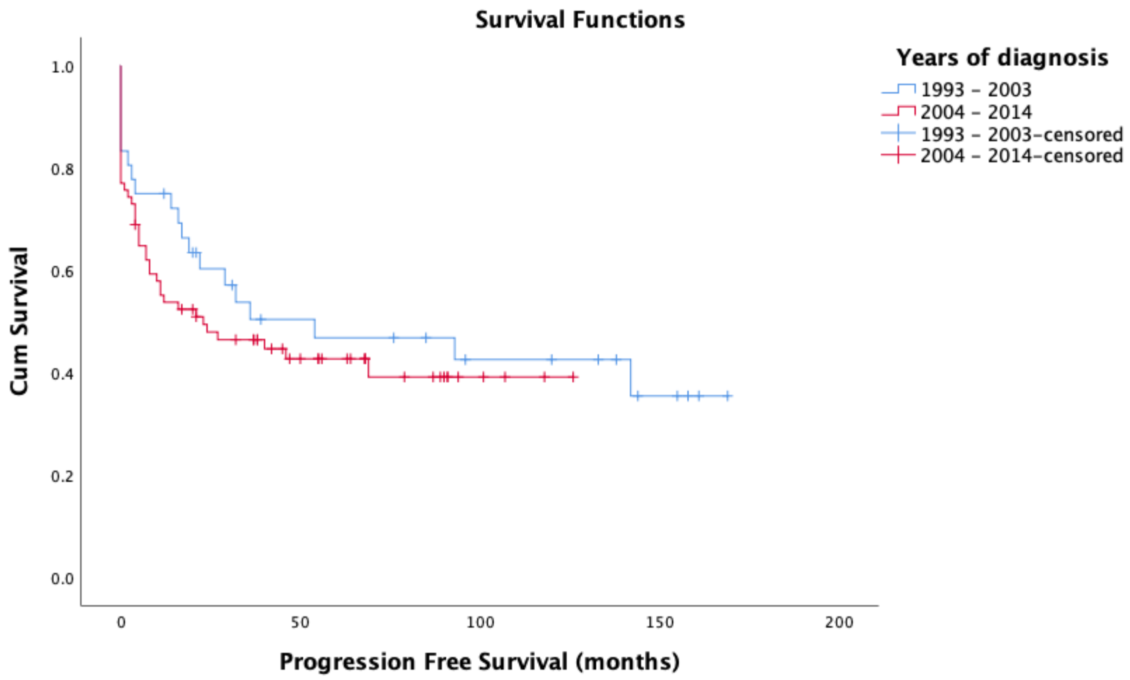

c. Progression-free survival of 110 patients stratified by the years of diagnosis

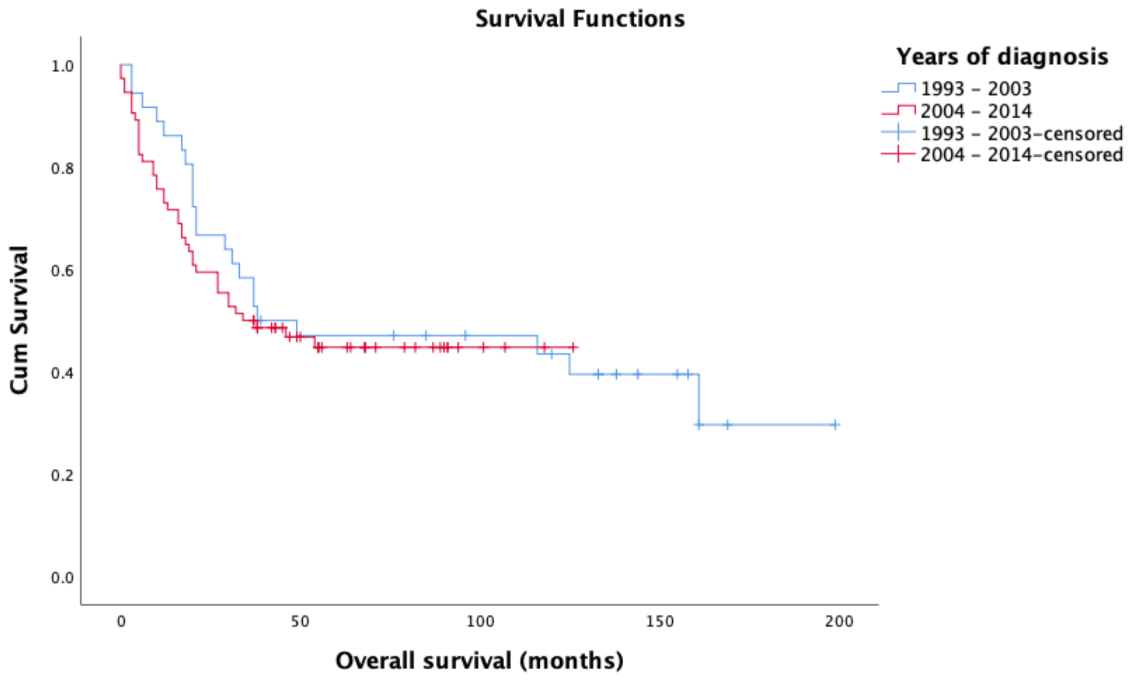

d. Overall survival of 110 patients stratified by the years of diagnosis

Figure S2 Median progression-free survival and overall survival of the original 201 cohort and the 110 patients with complete information on the nine parameters, stratified according to the years of diagnosis
