# Supplementary material for: Development of a Multi-Institutional Prediction Model for Three-Year Survival Status in Patients with Uterine Leiomyosarcoma (AGOG11-022/QCGC1302 Study)
Source: Cancers (Basel). 2021 May 14;13(10):2378. doi: 10.3390/cancers13102378 (PMC8155866; doi:10.3390/cancers13102378)
Supplement: Supplementary file 1 [file cancers-13-02378-s001.zip › Table S2_Survival data of the patients stratified by the years of diagnosis .pdf]

	1993 – 2003	2004 – 2014	P value
**201 patients**	69	132	
Median PFS (months)	33.0 (1.37 – 64.6)	39.0 (2.97 – 75.0)	0.970
Median OS (months)	40.0 (19.2 – 60.8)	60.0 (27.3 – 92.7)	0.986
Number of survival at 3rd year	38 / 66 (57.6%)	63 / 113 (55.8%)	0.812
**110 patients**	36	74	
Median PFS (months)	54.0 (0 – 129.5)	23.0 (0 – 54.8)	0.410
Median OS (months)	38.0 (0 – 129.0)	34.0 (5.2 – 62.8)	0.576
Number of survival at 3rd year	21 / 36 (38.3%)	37 / 74 (50.0%)	0.411

Table S2 Survival data of the original 201 cohort and the 110 patients with complete information on the nine parameters, stratified according to the years of diagnosis
